# A fatal familial insomnia patient newly diagnosed as having depression: A case report

**DOI:** 10.1097/MD.0000000000027544

**Published:** 2021-10-15

**Authors:** Tan Yukang, Liang Jiaquan, Li Xiaoling, Liu Yiliang, Xu Guohong, Xu Caixia, Xie Guojun

**Affiliations:** Department of Psychiatry, The Third People's Hospital of Foshan, Guangdong, People's Republic of China.

**Keywords:** clinical symptoms, fatal familial insomnia, imaging data, prion protein, prion protein examination

## Abstract

**Introduction:**

Fatal familial insomnia (FFI) is a rare clinical case. The study was mainly to report the clinical symptoms and imaging and genetic characteristics of a FFI case with depression, with relevant literature summarized.

**Patient concerns:**

A male, aged 57 years old, with mental disorders and progressive memory decline one year before admission.

**Diagnosis:**

Clinical manifestations: he had obvious abnormal mental behavior, rapidly progressing dementia symptoms, stubborn insomnia, abnormal movements and laryngeal stridor after falling asleep at night. Imaging and genetic test results: the cranial magnetic resonance imaging showed frontal temporal lobe atrophy; the polysomnography results showed no effective sleep; the 14-3-3 test result of cerebrospinal fluid was negative; the prion protein (PRNP) test showed that the D178N gene locus had mutations. And the patient was finally diagnosed as FFI.

**Interventions:**

There were no obvious effects in the treatment using medicines such as Risperidone, Olanzapine, Alprazolam, Clonazepam, and Deanxit.

**Outcomes:**

Mobility dysfunction of the patient was further aggravated. He was no longer able to move around on his own, and there were serious mental disorders.

**Conclusion:**

PRNP examination is of guiding significance for the diagnosis of the FFI of depression. Hence, it is very necessary to perform PRNP examination in clinical diagnosis of FFI of depression.

## 1 Introduction

Fatal familial insomnia (FFI) is a kind of hereditary prion protein (PRNP) diseases. Currently known common PRNP diseases are Creutzfeldt-Jakob disease, Kuru disease syndrome, and FFI.^[1]^ FFI is also a rare long chromosome mutation disease. The PRNP diseases initially manifest as dementia and dyskinesia, and there is no effective treatment for the disease at present.^[2]^ Studies on its etiology have found that the variant PRNP uses itself as a template to change the high-level results of normal PRNP and replicate them in large quantities, resulting in a large amount of variant PRNP accumulating in nerve cells and ultimately leading to nerve cell death and glial cell proliferation.^[3]^ FFI, as the rarest kind of PRNP disease, was first reported by foreign scholars in 1986, and there have been many reports about it from abroad since then.^[4]^ The first case was reported in 200in China, and many cases have been reported since then. There are still not many reports on the disease currently. Consequently, the pathogenesis is still not very clear.^[5]^ With the continuous development of molecular biology, some scholars are trying to study the disease from the molecular and protein level.

FFI is clinically believed to be generally manifested as refractory insomnia, dyskinesia, and endocrine changes. The main pathological features are massive loss of thalamic neuronal cells and glial cell proliferation. PRNP test results mostly show that the D178N gene locus has mutations.^[6]^ For example, an Italian scholar conducted a study on 3 FFI patients in the same family. It was found that all 3 patients had dyskinesia and sleep disorders, which progressed rapidly, and all 3 patients died within 5 to 10 days after the onset. Neuropathological examination of the 3 patients all found degeneration of the thalamus and olive nucleus, and gene sequencing revealed the PRNP gene 178 mutation.^[7]^ However, there are also reports that the main clinical manifestations of some FFI patients are not consistent with those mentioned above. For example, it was noted that there were FFI cases with Biot's respiration as the main manifestations.^[8]^ Some scholars conducted research on cases of dementia that were misdiagnosed as Alzheimer's disease, and then they were further diagnosed as having FFI through genetic testing. Some scholars have conducted comparative studies on FFI and human Creutzfeldt-Jakob disease, both belonging to PRNP diseases. Both PRNP diseases are related to D178 mutations. If the PRNP allele C129 is methionine, it is fatal insomnia, and if C129 is valine, it is human Creutzfeldt-Jakob disease.^[9]^ Taken together, there are no accurate and comprehensive diagnostic criteria for FFI, which is not conducive to the diagnosis and treatment of FFI. In recent years, domestic scholars have conducted research on the PRNP gene of the Chinese mainland population. They found that compared with the European population, the frequency of the 129 M/M gene in the Han, Uighur, and Hui populations in China is significantly higher, suggesting that the Chinese population is more likely to be infected with PRNP. Based on the above research, it is concluded that different races may have distinct susceptibility to FFI, clinical symptoms, pathological test results, and genetic test results.^[10]^ At present, there are few reports about the disease in China. In other words, the research data of the disease is extremely lacking, seriously hindering the research progress of FFI in China. In the study, an FFI patient with depression, treated in the Third People's Hospital of Foshan, was selected as the research subject, with the related literature summarized. It is hoped that it can provide a basis for the research of FFI and promote the development of research on this disease in China.

## 2 Methods

### 2.1 Research subjects

An FFI patient treated in the Third People's Hospital of Foshan in March, 2020 with mental abnormalities as the main complaint were selected as the research subjects, as well as his families. The patient was a male, aged 57 years old, with mental disorders and progressive memory decline 1 year before admission. After admission, his mental and cognitive condition deteriorated progressively and died a few months later. His imaging data and clinical characteristics during the hospitalization period were collected. The PRNP examination was performed on the patient and his families for family analysis. Before the experiment, the patient and his immediate family members signed informed consent. The study protocol was approved by the Ethics Committee at the Third People's Hospital of Foshan, China.

### 2.2 Family investigation

The family investigation of the proband was completed by pedigree analysis. The specific operations were as follows. With the proband as the entry point, some information was traced including the number of all family members, kinship relationships, and the distribution of certain genetic diseases or traits. The information was then sorted and displayed in the form of a map.

### 2.3 Extraction of peripheral blood DNA

2 mL of peripheral blood was drawn from the proband, his mother, sister, and daughter, respectively, added to an anticoagulation test tube containing sodium citrate. After gently shaken and mixed, 0.3∼0.5 mL was transferred to 1 mL of purified resin, followed by the inverted mixing operations 5 to 6 times. Subsequently, it was incubated at room temperature for 3 minutes. During this period, it was mixed with upside down once, and then centrifuged at 5000 rpm for 3 seconds, with the precipitate collected. The purified resin was suspended with 1 mL of GN binding solution. After mixed uniformly, it was centrifuged at 5000 rpm for 3 seconds, with the precipitate collected. The purified resin was then washed twice with 0.5 mL of rinsing solution. After mixed evenly, it was centrifuged at 5000 rpm for 3 seconds, with the precipitate collected. After suspended with 0.8 mL of absolute ethanol, the mixture was put in a purification spin column, followed by centrifugation at 12000 rpm for 1 minute, with the ethanol in the waste liquid collection tube discarded. Another centrifugation operation was performed to completely remove the ethanol as much as possible. The centrifugal purification column was put in a 1.5 mL centrifuge tube, with 100uL TE buffer added. After let still at room temperature for 3 minutes, it was centrifuged at 12000 rpm for 2 minutes. The liquid in the centrifuge tube was the eluted genomic DNA, and 2 uL was then drawn for electrophoresis. The electrophoresis conditions were: 1% agarose, 120 V, 20 minutes.

### 2.4 Polymerase chain reaction amplification

**Figure 1:**
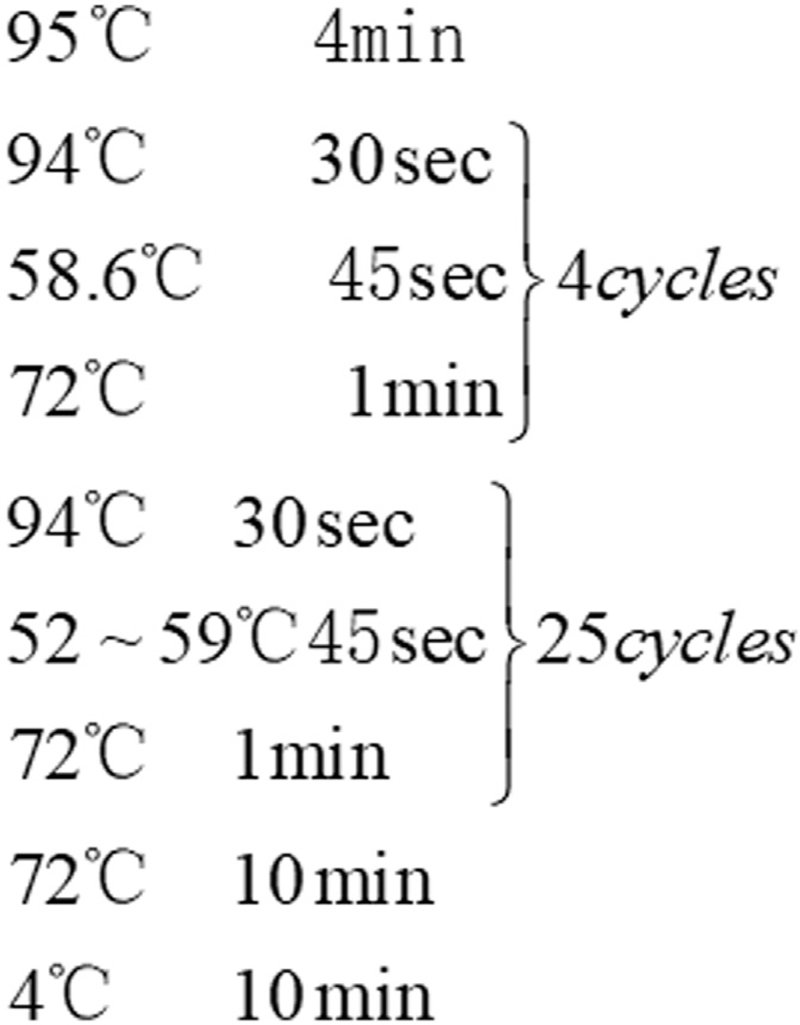
The specific Polymerase Chain Reaction (PCR) circulation procedure.

The Polymerase Chain Reaction (PCR) amplification system consisted of template Plasmid 2.5 μL, 10 ×Taq Buffer 5 μL, primer FLPS 2.5 μL, primer FLPA 2.5 μL, Taq enzyme 2.5 μL, MgCl_2_ 2 μL, and dNTP 5 μL, with ddH_2_O used to keep the volume to 50 μL. There were 5 tubes in total, each tube had a volume of 10 μL, and the temperature of each tube was set to 53.1°C, 54.3°C, 54.8°C, 57.2°C, 59.0°C, respectively. The specific PCR circulation procedure was shown in Figure 1.

### 2.5 Polymerase chain reaction product identification

The agar gel containing 1.2% ethidium bromide was prepared for electrophoresis, together with 2 uL PCR product and 1 uL loading buffer, with 100 bp DNA ladder as Marker. The electrophoresis lasted for 40 minutes under 90 V voltage and 40 mA current. When the indicator reached about 2/3 of the gel, the electrophoresis was finished. After electrophoresis, the PCR amplification results were observed under a UV light.

### 2.6 Purification and sequencing of polymerase chain reaction products

The sequencing and purification of PCR products were all completed by the Viral Disease Control and Prevention Institute of the Chinese Center for Disease Control and Prevention. All samples were sequenced in both directions, and the sequencing results of all amplified products were completely consistent. After the sequencing, the sequencing results were compared with the standard sequence published on Pubmed to determine the mutation loci and the changes in the encoded amino acids sequence. With the mutation loci determined, PCR amplification was performed on of the corresponding loci of his family members, followed by purification and analysis.

## 3 Case report

### 3.1 Clinical manifestations

The patient was 57 years old and had abnormal mental state and sleep behaviors at night after admission. He often only fell asleep for 2 to 3 hours, and it was difficult to fall asleep again after waking up. As a result, he failed to fall asleep all night. The family members reported that the patient's limbs often kept groping after falling asleep. He was often listless during the day, and easily fell asleep while sitting or lying down. His ability to use chopsticks declined rapidly, and walking and eating disorders appeared then. When the patient just woke up, he said that a relative came to visit him. However, his family members reported that no one came to visit at that time. A few months later, the patient's mental and cognitive condition deteriorated more severely, and he was unable to walk independently and needed the support of others to move around. During the hospitalization, the neuropsychological test results of the patient were as follows. Social Disability Screening Schedule indicated that the patient has mild social function impairment; Mini-mental State Examination score of 13 points indicated that the patient has moderate mental function impairment; Hamilton Anxiety Scale score of 15 points indicated that the patient was anxious; Hamilton Depression Scale score of 20 indicated that the patient has mild depression. The patient can not cooperate with the doctor in mini-mental state examination and Montreal Cognitive Assessment examinations as a result of the inability to speak autonomously. The Activity of Daily Living scale score was 70 points (>22 points were abnormal). The Neuropsychiatric Inventory results showed that the abnormal mental behaviors of the patient in the past month mainly included agitation, anxiety, apathy, disinhibition, confusing behavior, sleep disorders, and changes in mouth desire. The Clinical Dementia Rating scale score was 3 points (normal: 0 points, suspicious dementia: 0.5 points, mild dementia: 1 point, moderate dementia: 2 points, severe dementia: 3 points). Polysomnography revealed that: his sleep time was about 1 hour. Besides, short onset Rapid Eye Movement period, respiratory obstruction, hypopnea, excessive Rapid Eye Movement sleep, and short-term myoelectric activity were noted.

### 3.2 The family investigation

**Figure 2:**
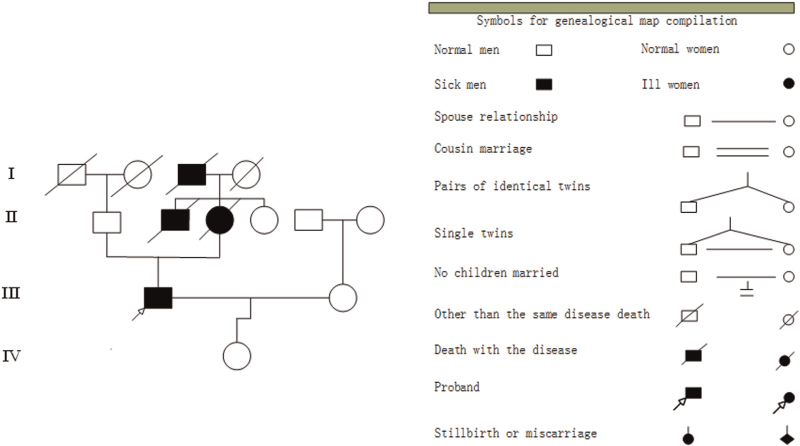
The family tree of the proband.

In this study, a total of 13 members of the 4 generation were investigated. The specific family map was shown in Figure 2. Seven of the 13 people died, including the proband. There were 3 patients with suspected FFI, one of which was the mother of the proband. The grandfather of the proband died earlier, and the cause of death could not be verified. However, according to the description of families, the grandfather had similar clinical symptoms, but no pathological and genetic examinations were performed. The father of the proband did not undergo genetic testing due to loss of contact.

### 3.3 Magnetic resonance imaging results

**Figure 3:**
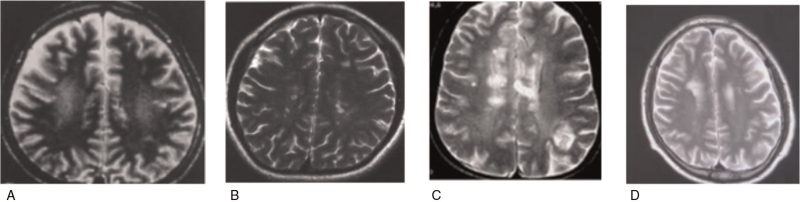
The cranial Magnetic resonance imaging (MRI) results.

The cranial magnetic resonance imaging examination results of were shown in Figure 3. Figure 3A suggested lateral frontal lobe atrophy; Figure 3B suggested lateral sacral lobe atrophy, and widened lateral fissure and the third ventricle; Figure 3C indicated bilateral hippocampal atrophy; Figure 3D were the re-examination T1WI results, suggesting bilateral frontal lobe atrophy.

### 3.4 Polymerase chain reaction results

**Figure 4:**
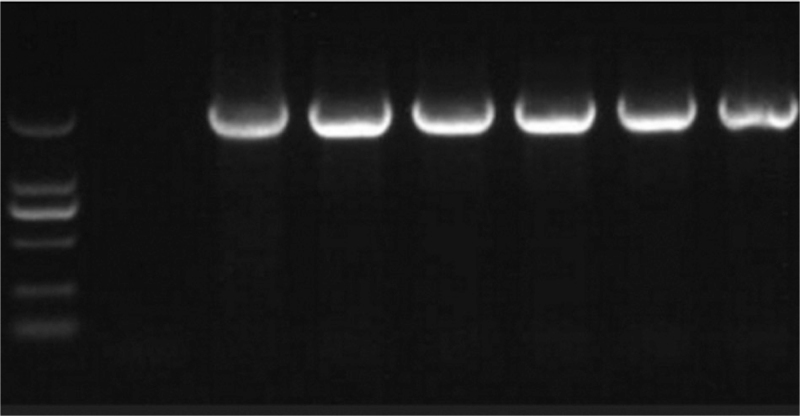
Amplified bands at different temperatures.

**Figure 5:**
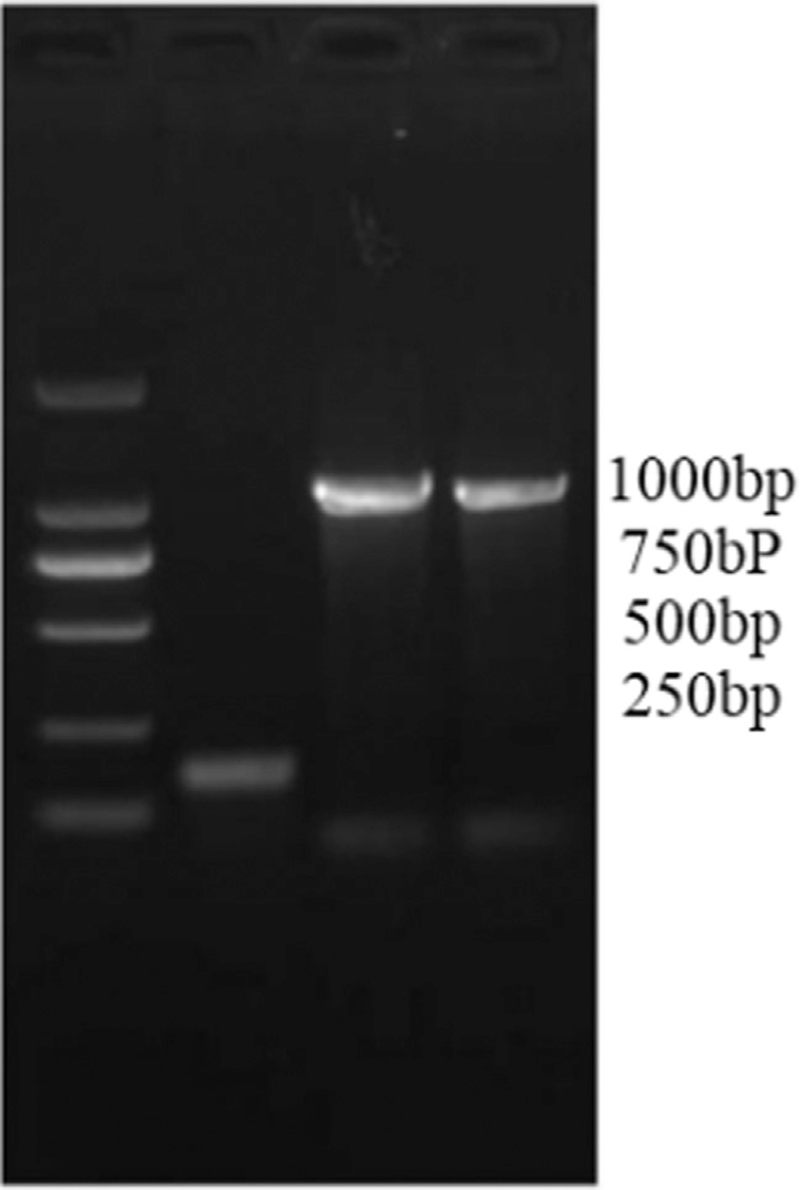
The size of the amplified Prion protein (PRNP) gene fragment.

The PCR amplification results were shown in Figure 4 and Figure 5. It was evident from Figure 4 that the amplified fragments were consistent with the target fragments, and each experiment was repeated twice. It was evident from Figure 5 that the length of the amplified PRNP gene fragment was 1000 bp.

### 3.5 Purification and sequencing results of polymerase chain reaction products

**Figure 6:**
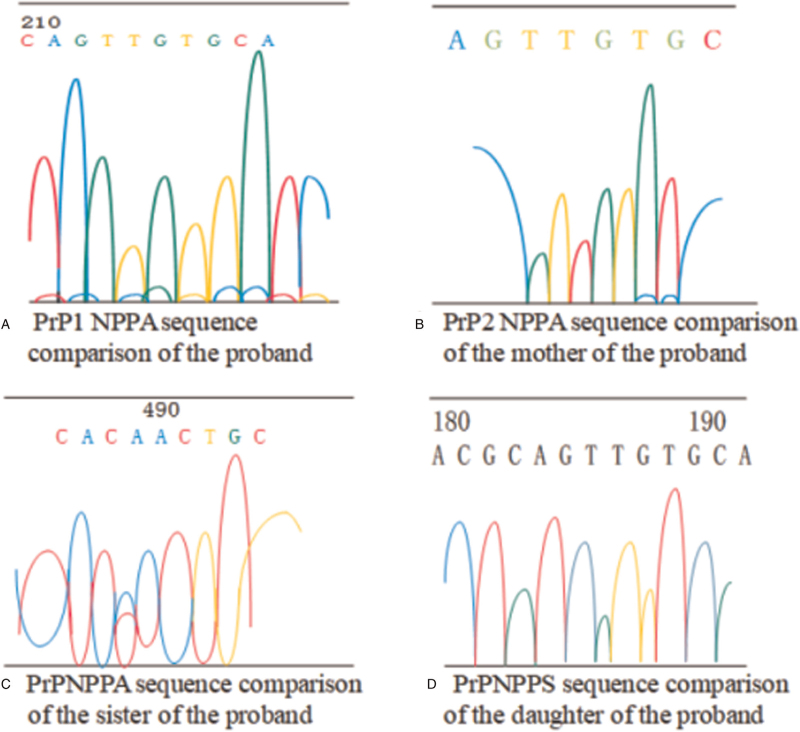
The reverse sequencing of the PRNP amplified gene on chromosome 20 of the proband and his three families.

Figure 6 showed the reverse sequencing of the PRNP amplified gene on chromosome 20 of the proband and his three families (mother, sister, daughter). According to Figure 6, the 532nd base of the PRNP gene coding region of the proband's chromosome 20 was mutated from guanine (G) to adenine (A), which further caused the 178th amino acid of PRNP to be mutated from aspartic acid to asparagine. It was evident from Figure 6 that no sample of the patient's 3 families was detected of PRNP gene mutations, the 129 amino acid polymorphism was of the M/M type, and that of the 219 amino acid was of E/E type.

### 3.6 Treatment, outcome and follow-up information

There were no obvious effects in the treatment using medicines such as Risperidone, Olanzapine, Alprazolam, Clonazepam, and Deanxit, and the mental, cognitive, and mobility dysfunction of the patient was further aggravated. He was no longer able to move around on his own, and there were serious mental disorders such as going to the toilet without lifting their pants and throwing toothpaste into the toilet for no reason.

## 4 Discussion

At present, there is no unified standard for the diagnosis of FFI worldwide. The mainstream view is that the age of onset is mostly around 50, and the course of disease is mostly 7 to 72 months. In the study, FFI cases that have been reported in China were collected and analyzed. It was found that the age of onset of each case was 22 years, 33 years, 54 years, 49 years, and 32 years old, respectively, with an average onset age of 38 years; the course of disease was 7 months, 13 months, 11 months, 37 months, and 7 months, respectively, with an average course of disease of 15 months.^[11]^ The age of onset of the subject in this study was 43 years old, and the course of disease was 10 months, consistent with the general age of onset and course of disease in China. It is generally believed that FFI is initially manifested as mild sleep disorder, reduced sleep time, decreased sleep quality, difficulty falling asleep at night, easy to be awakened, and difficulty falling asleep again after waking,^[12]^ which can not be relieved by hypnotics. With the worsening of the disease, the sleep quality of the patient further declines. After falling asleep at night, they often exhibit abnormal behaviors such as moving around, laryngeal stridor, and biting. The patient will then experience cognitive dysfunction and dyskinesia as a result of further worsened condition. Common symptoms are hallucinations, to soliloquize, low mood, slow response, and inability to act on their own.^[13]^ However, there are also patients whose clinical symptoms are not consistent with those above. For example, it is noted that there are FFI cases with Biot's respiration as main manifestations.^[14]^ The case in this study is a typical patient with insomnia and decreased sleep quality as the initial symptoms, and sleep disorder was progressively worsening. In the later stage, abnormal behaviors such as moving around, biting the quilt, laryngeal stridor, and raving gradually appeared after he fell asleep at night. During the daytime, he was depressed and unresponsive, and easy to fall asleep when sitting, standing, and lying in bed. After waking up, he often talked to himself, saying that he had relatives who came to visit him, but there was no one visiting him at that time.

FFI is a genetic PRNP disease with autosomal mutations. The discovered PRNP diseases include human Creutzfeldt-Jakob disease, Kuru disease syndrome, and FFI. Among them, FFI is the rarest PRNP disease,^[15]^ and there is currently no effective treatment for this disease. Studies on its etiology have found that the variant PRNP uses itself as a template to change the high-level results of normal PRNP and replicate them in large quantities, resulting in a large amount of variant PRNP accumulating in nerve cells and ultimately leading to nerve cell death and glial cell proliferation.^[16]^ It was first reported in 1986 by foreign scholars, and since then many cases have been reported abroad. The earliest report of the disease in China was in 2004, and many cases have been reported since then. There are still not many reports on the disease currently. Consequently, the pathogenesis is still not very clear.^[17]^ With the continuous development of molecular biology, some scholars are trying to study the disease from the molecular and protein level.

The PRNP test of FFI reveals that the D178N gene locus has mutations, accompanied by nerve cell death and glial cell proliferation. For example, an Italian scholar conducted a study on 3 FFI patients in the same family. It was found that all 3 patients had dyskinesia and sleep disorders, which progressed rapidly, and all 3 patients died within 5 to 10 days after the onset. Neuropathological examination of the 3 patients all found degeneration of the thalamus and olive nucleus, and gene sequencing revealed the PRNP gene 178 mutation.^[18]^ For example, some scholars have studied cases of dementia that were misdiagnosed as Alzheimer's disease, and then they were further diagnosed as having FFI through genetic testing. Some scholars have conducted comparative studies on FFI and human Creutzfeldt-Jakob disease, both belonging to PRNP diseases. Both PRNP diseases are related to D178 mutations. If the PRNP allele C129 is methionine, it is fatal insomnia, and if C129 is valine, it is human Creutzfeldt-Jakob disease.^[19]^ In recent years, domestic scholars have conducted research on the PRNP gene of the Chinese mainland population. They found that compared with the European population, the frequency of the 129 M/M gene in the Han, Uighur, and Hui populations in China is significantly higher, suggesting that the Chinese population is more likely to be infected with PRNP than European population.^[20]^ Based on the above research, it is concluded that different races may have distinct susceptibility to FFI, clinical symptoms, pathological test results, and genetic test results. In this study, it was found that the 532nd base in the coding region of the PRNP gene on chromosome 20 of the proband mutated from guanine (G) to adenine (A), which further caused the 178th amino acid of PRNP to be mutated from aspartic acid to Asparagine. This illustrated that the N178 mutation was highly associated with FFI, consistent with the research results at worldwide. However, this case did not have offspring, and the research results needed to be further improved.

Therefore, it is noted that the etiology of FFI is complex, leading to difficulty in treatment. At the same time, there is little research data in China. Although foreign countries are ahead of us in the research of this disease, the clinical symptoms and case characteristics may be different due to factors such as ethnic differences. Consequently, it is reasonable to learn from research results and treatment experience abroad instead of copying them. In this study, the related information of an FFI case, treated in the Third People's Hospital of Foshan, was collected for clinical manifestations, pathological features, and imaging data, with genetic testing and family studies carried out at the same time. It is expected to provide a basis a for the research of FFI in China. However, due to limited sample size, this study is not in-depth and comprehensive enough, and further research is needed.

## Author contributions

**Data curation:** Liang Jiaquan.

**Formal analysis:** Liang Jiaquan.

**Funding acquisition:** Li Xiaoling, Xu Guohong, Xu Caixia.

**Investigation:** Li Xiaoling.

**Methodology:** Li Xiaoling, Xie Guojun.

**Project administration:** Xu Guohong, Xie Guojun.

**Resources:** Liang Jiaquan.

**Software:** Liang Jiaquan, Liu Yiliang.

**Supervision:** Xu Caixia, Xie Guojun.

**Validation:** Xu Caixia.

**Visualization:** Xu Caixia.

**Writing – original draft:** Liang Jiaquan.

**Writing – review & editing:** Tan Yukang.
